# Mitochondrial transfer from mesenchymal stem cells to neural stem cells protects against the neurotoxic effects of cisplatin

**DOI:** 10.1186/s40478-018-0644-8

**Published:** 2018-12-12

**Authors:** Nabila Boukelmoune, Gabriel S. Chiu, Annemieke Kavelaars, Cobi J. Heijnen

**Affiliations:** Laboratory of Neuroimmunology, Department of Symptom Research, The University of Texas MD Anderson Cancer Center, 1515 Holcombe Blvd, Unit 384, Houston, TX 77030 USA

**Keywords:** Mesenchymal stem cells, Neuronal stem cells, Mitochondrial transfer, Cisplatin

## Abstract

Mesenchymal stem cells (MSCs) transfer healthy mitochondria to damaged acceptor cells via actin-based intercellular structures. In this study, we tested the hypothesis that MSCs transfer mitochondria to neural stem cells (NSCs) to protect NSCs against the neurotoxic effects of cisplatin treatment. Our results show that MSCs donate mitochondria to NSCs damaged in vitro by cisplatin. Transfer of healthy MSC-derived mitochondria decreases cisplatin-induced NSC death. Moreover, mitochondrial transfer from MSCs to NSCs reverses the cisplatin-induced decrease in mitochondrial membrane potential. Blocking the formation of actin-based intercellular structures inhibited the transfer of mitochondria to NSCs and abrogated the positive effects of MSCs on NSC survival. Conversely, overexpression of the mitochondrial motor protein Rho-GTPase 1 (Miro1) in MSCs increased mitochondrial transfer and further improved survival of cisplatin-treated NSCs.

In vivo, MSC administration prevented the loss of DCX+ neural progenitor cells in the subventricular zone and hippocampal dentate gyrus which occurs as a result of cisplatin treatment. We propose mitochondrial transfer as one of the mechanisms via which MSCs exert their therapeutic regenerative effects after cisplatin treatment.

**Electronic supplementary material:**

The online version of this article (10.1186/s40478-018-0644-8) contains supplementary material, which is available to authorized users.

## Introduction

Mesenchymal stem cells (MSCs) have been shown to stimulate tissue repair in various disease models, including cardiomyopathy, pulmonary damage, cerebral ischemic insults, and neurodegenerative disorders like Alzheimer and Parkinson disease [8]. Multiple mechanisms have been proposed to mediate these beneficial effects of MSCs, including suppression of inflammation and release of growth factors. Recently, it has become apparent that transfer of healthy mitochondria to damaged cells represents an important mechanism of endogenous regeneration. For example, astrocytes have been shown to transfer mitochondria to neurons after ischemic stroke in mice [15]. MSCs also transfer mitochondria to cardiomyocytes in a model of anthracycline-induced cardiomyopathy [46], to murine alveoli in acute lung injury [17], to murine lung epithelial cells in rotenone-induced airway injury [2], to cortical neurons in a model of cerebral stroke [5] and to human monocyte-derived macrophages as well as murine alveolar macrophages in models of acute respiratory distress syndrome [18].

Platinum-based chemotherapeutic agents, such as cisplatin, are widely used to treat solid tumors [19]. Increasing evidence indicates that cognitive deficits develop in cancer patients treated with chemotherapy. Cognitive deficits induced by cancer treatment are characterized by confusion, memory loss, reduced attention and processing speed, and decreased executive functioning [20–22, 39, 43]. Longitudinal neuropsychological studies report that up to 75% of cancer patients experience cognitive problems during treatment and likely 35% of affected cancer patients have long-term cognitive effects that seriously impair their quality of life [1].

We recently reported that cisplatin induces cognitive impairment, synaptosomal mitochondrial dysfunction, and changes in neuronal mitochondrial morphology in mice [7]. Nasal administration of MSCs to cisplatin-treated mice restored cognitive function and normalized mitochondrial function [6]. Lomeli et al. have demonstrated that MSCs reduce cranial irradiation-induced brain damage as well [28].

Neurogenic precursor proliferation and differentiation, particularly in the dentate gyrus (DG) of the hippocampus, is crucial to formation of new neurons, thereby enhancing neural circuity and improving learning and memory [37, 41, 47]. Disruption of adult neurogenesis contributes to the pathogenesis of many neurodegenerative diseases associated with cognitive impairment. In vivo and in vitro, cisplatin induces loss of neuronal precursors [3, 7, 28]. Gong el al. have shown that neuronal precursors are very sensitive to low cisplatin concentrations [13].

The aim of the present study was to test the hypothesis that MSCs can protect neuronal stem cells (NSCs) in culture, in vitro against the neurotoxic effects of cisplatin through mitochondrial donation. Moreover, we investigated whether nasal administration of MSCs in vivo protects the brain neurogenic pools against the damaging effects of cisplatin.

## Materials and methods

### Animals

9 weeks old C57BL/6 J male mice (Jackson Laboratory) were used. Mice were housed on a 12/12 h reverse dark–light cycle. Animals had access to water and food ad libitum. All experiments were conducted at The University of Texas MD Anderson Cancer Center in Houston, Texas. Animals were used in accordance with Institutional Animal Care and Use Committee-approved protocols.

### Cell culture and transfection

Mouse cortical NSCs (R&D Systems, Minneapolis, MN, USA) were cultured in monolayers in low-glucose Dulbecco’s Modified Eagle’s Medium (DMEM)/F12, supplemented with 100 U/mL penicillin and N-2 plus supplement (R&D Systems). Fibroblast growth factor basic and epidermal growth factor (both 20 ng/mL; R&D Systems) were added to the cultures daily. NSCs were cultured on surfaces coated with poly-L-ornithine (Sigma-Aldrich, St. Louis, MO, USA) and bovine fibronectin (R&D Systems) and detached using Accutase (Innovative Cell Technologies, San Diego, CA, USA).

C57BL/6 mouse MSCs (Invitrogen) were grown in 5% CO_2_ at 37 °C in DMEM/F12 medium with GlutaMax-I, supplemented with 10% MSC-qualified fetal bovine serum and 5 μg/mL gentamycin (all from GIBCO, Carlsbad, CA, USA). Before transfection, MSCs were seeded on plates coated with poly-L-lysine (Sigma-Aldrich). Cells were harvested using TrypLE-express (GIBCO). For labeling mitochondria we used pLYS1-FLAG-MitoGFP-HA (Addgene plasmid # 50057; gift from Vamsi Mootha [35]) which contains the pore-forming subunit of the mitochondrial calcium uniporter to target the label to the mitochondria. The mito-mcherry plasmid was generated by cloning the mito-GFP insert of the pLYS1-FLAG-MitoGFP-HA plasmid into the mcherry2-N1 vector (Addgene plasmid # 54517, gift from Michael Davidson). The mitochondrial rho GTPase 1 (Miro1)-GFP overexpression plasmid (RhoT1, MG224107) was obtained from Origene (Rockville, MD, USA). Transfection of mito-GFP, mito-mcherry and Miro1-GFP plasmids were performed using either Lipofectamine LTX (Invitrogen) with Plus reagent for mito-GFP or mito-mcherry expression in MSCs or Lipofectamine 2000 (Invitrogen) and JetPrime (Polyplus Transfection, New York, NY, USA) for Miro1 overexpression, according to the manufacturer’s instructions.

### Chemotherapy and MSC treatment

Cisplatin (2.3 mg/kg/day; Teva, Petah Tiva, Israel) or phosphate-buffered saline (PBS) was administered daily to mice intraperitoneally, following a 5 days injections and 5 days rest scheme. Prior to MSC application, mice received 3 μl of hyaluronidase in PBS in each nostril (100 U per mouse, Sigma-Aldrich) to increase the permeability of the nasal mucosa [6, 10, 11, 16]. 30 min after, 3 μl of MSC cell suspension (1 × 10^6^ cells per mouse per day) or PBS were administered to mice, twice in each nostril, for a total of 12 μl. MSCs were applied 48 and 96 h after the last cisplatin injection.

### Co-culture of MSCs and NSCs

NSCs (35 × 10^4^ cells) were plated on cell culture imaging dishes (ibidi, Fitchburg, WI, USA). Two days after plating, NSCs were treated with cisplatin (Teva, Petah Tikva, Israel) for 8 h, stained with 20 μM CellTracker Blue fluorescent probe (CTB; Invitrogen, Carlsbad, CA) for 45 min at 37 °C, and washed in serum-free media. MSCs (15 × 10^4^ cells) were added to the culture for 17 h. Co-cultures were stained with wheat germ agglutinin (WGA) conjugates (WGA 488 or WGA 594, 1/300 dilution; Invitrogen) for 10 min at 37 °C, followed by 2 washes in Hank’s Balanced Salt Solution (GIBCO) prior to imaging in Live Cell Imaging Solution (Invitrogen).

To inhibit actin polymerization, MSCs were treated with 2 μM Latrunculin B (Sigma-Aldrich) for 24 h prior to co-culture with NSCs. To assess NSC survival, CTB-positive NSCs were counted using the countess II FL automated cell counter (Invitrogen). Mitochondrial transfer was quantified in representative confocal images of every condition used, and the percentage of either mito-GFP-positive or mito-mcherry-positive NSCs was determined.

### Analysis of mitochondrial membrane potential

After exposure to cisplatin and co-culture with MSCs, cells were stained with 250 nM of the fluorescent mitochondrial membrane potential-sensitive dye tetramethylrhodamine methyl ester (TMRM, Invitrogen) for 45 min at 37 °C. As a positive control, NSCs were treated with 10 μM carbonilcyanide p-triflouromethoxyphenylhydrazone (FCCP), a mitochondrial uncoupler, for 15 min. Cells were imaged by confocal microscopy or washed with serum-free media and collected in a single cell suspension for flow cytometry analysis. The fluorescence signal was detected with a BD accuri C6 Flow Cytometer (BD Biosciences, San Jose, CA USA) at FL2 emission of 585/40 nm. We used the TMRM in sub-quench mode, as described previously [30, 33].

### Analysis of mitochondrial bioenergetics

To assess mitochondrial bioenergetics, NSCs were grown in a Seahorse XFe 24 microplate (Seahorse Biosciences/Agilent Technologies, Santa Clara, CA, USA) coated with Poly-L-Ornithine and fibronectin to 80% confluency. NSCs were treated with 0.5–1 μM cisplatin or vehicle for 12 h, washed with serum-free media, and incubated for 1 h at 37 °C in XF base media (Seahorse Biosciences) supplemented with 11 mM glucose, 2 mM glutamine, and 1 mM pyruvate. Oligomycin (2 mM), FCCP (4 mM), and rotenone/antimycin A (2 mM each) were used with a 3-time repeat of a 2-min mix, 3-min wait, and 2-min measure assay cycle. Oxygen consumption rates were normalized to the total protein content of each well. Basal respiration, adenosine triphosphate (ATP)-linked respiration, proton leak, and maximal respiratory capacity were determined as described previously [7, 23].

### Immunohistochemistry

Immunostaining of neural precursors in the dentate gyrus (DG) and subventricular zone (SVZ) was performed as previously reported [7]. Briefly, mice were euthanized and perfused with PBS followed by 4% paraformaldehyde (PFA). Brains were removed and fixed in 4% PFA for 48 h, paraffin embedded and sectioned at 8 and 10 μm for the SVZ and DG, respectively. Brain slices were stained for doublecortin (DCX, 1:50; Abcam, Cambridge, UK) followed by AlexaFluor 488 Donkey secondary antibody (Invitrogen) and DAPI. Sections were imaged and positive staining was quantified using ImageJ. The number of DCX+ cells was quantified in representative images by researchers blinded to treatment.

To characterize NSCs, immunostaining was done as previously described [4]. Briefly, cells were fixed with 4% paraformaldehyde in PBS, treated with 0.25% Triton X-100, blocked in 2% BSA in PBS and stained with either anti-DCX antibody (1:50; Abcam, Cambridge, UK), anti Sox2 (1/200; Millipore, Burlington, MA), anti βIII-Tubulin (1/200; R&D Systems) or anti Nestin (1/1000, Abcam) and anti-GFAP (1/200, Acris, Rockville, MD) in blocking buffer followed by secondary antibody and DAPI. NSCs used for experiments were 52% Nestin+, 17% GFAP+, 16% Nestin/GFAP double-positive, 92% DCX+, 94% Sox2+ and 5% βIII-Tubulin+.

### Confocal microscopy

Live cell images of MSC and NSC co-cultures, fixed NSC staining as well as DCX expression in the DG and SVZ, were acquired using a SPE Leica Confocal Microscope (Leica Microsystems, Buffalo Grove, IL, USA) with a 63 X or 40 X objective, and analyzed with LAS X software. Image J was used to quantify TMRM intensity in individual cells. Data are expressed as the corrected total cell fluorescence = integrated density – (area of selected cell × mean fluorescence of background readings) [31].

### Statistical analysis

Data are presented as mean ± standard error of the mean (SEM) of at least 3 independent experiments. Data were analyzed using GraphPad Prism 7 (GraphPad Software, La Jolla, CA, USA). One-way or two-way analysis of variance (ANOVA) was used with or without repeated measure followed by either Bonferroni’s or Tukey’s correction for multiple comparisons, Dunn’s multiple comparison or using Student’s t-test, as appropriate and indicated in the legends.

## Results

### MSCs rescue NSC from cisplatin-induced cell death in vitro and in vivo

**Fig. 1:**
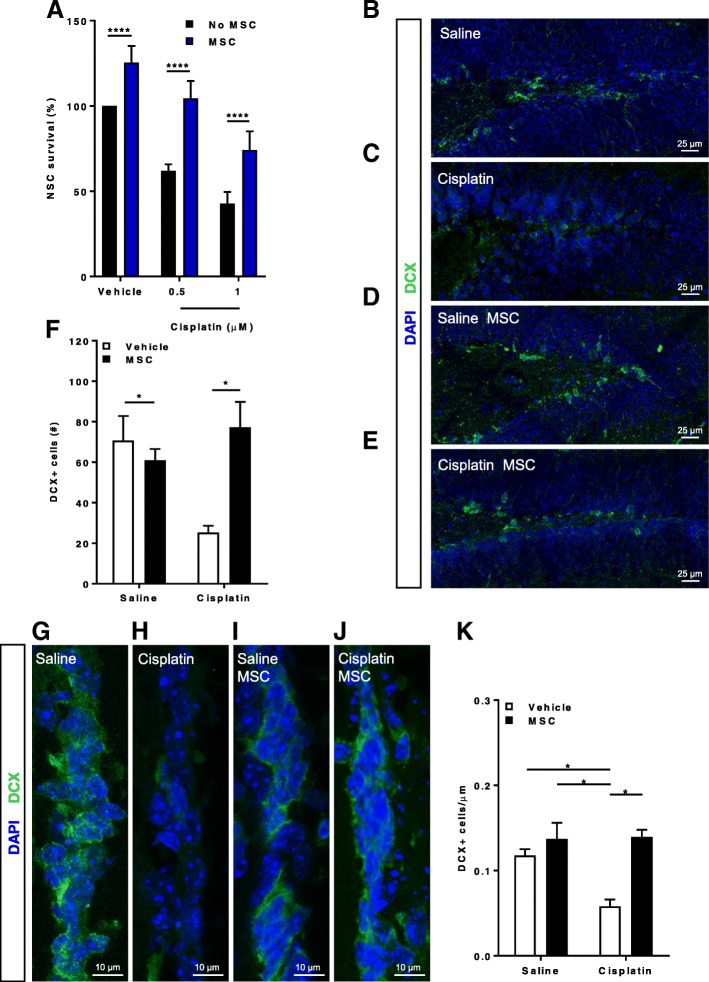
MSCs rescue damaged NSCs after cisplatin treatment and reverse the loss of neuroblasts in the brain. **a** Neuronal stem cells (NSCs) were treated with cisplatin or vehicle for 8 h, stained with cell tracker blue (CTB), and subsequently co-cultured for 17 h with or without mesenchymal stem cells (MSCs). Survival of NSCs was assessed by counting the number of CTB-positive cells. The graph shows the rate of NSC survival after 17 h co-culture with MSCs (blue bars) or without MSCs (black bars). Data are normalized to survival in the absence of MSCs and cisplatin in each experiment and represent the mean ± SEM of 6 independent experiments. Data were analyzed using two-way ANOVA, repeated measures (cisplatin × MSC interaction: *P* < 0.01), followed by Bonferroni’s post-hoc test. **** P < 0.0001). (**b**-**k**) Animals were treated with cisplatin for 2 cycles of 5 days. DCX+ neuronal progenitors were observed in the DG of the hippocampus (**b**-**e**) as well as the SVZ (**g**-**j**). The number of cells were counted in the DG tip (**f**). For the SVZ, the number of cells was normalized to the length of the SVZ (**k**). Data were analyzed by two-way ANOVA followed by Tukey’s post-hoc test. *P < 0.05

NSCs were treated with 0, 0.5 or 1 μM cisplatin for 8 h and stained with Cell Tracker Blue fluorescent probe. Subsequently, the Cell Tracker Blue positive neurons were co-cultured with MSCs for 17 h and recovery of the NSCs was quantified. Figure 1a shows that cisplatin dose-dependently reduced NSC survival. Addition of MSCs significantly increased survival of NSCs.

Next, we tested whether MSCs also rescue NSCs from cisplatin-induced cell loss in the DG of the hippocampus and the SVZ. Mice were injected with cisplatin during two cycles of 5 days (2.3 mg/kg) with 5 days of rest in between [7, 23]. One month after completion of cisplatin treatment, we observed a 40% decrease in the DCX+ neural progenitors in the DG of the hippocampus (Figs. 1f) and a 50% decrease in the SVZ (Fig. 1k). Nasal application of MSCs at 48 and 96 h after the last cisplatin dose reduced the cisplatin-induced loss of DCX+ neuronal progenitors in both the DG (Figs. 1f) and SVZ (Figs. 1k).

### Cisplatin induces mitochondrial damage in NSCs

**Fig. 2:**
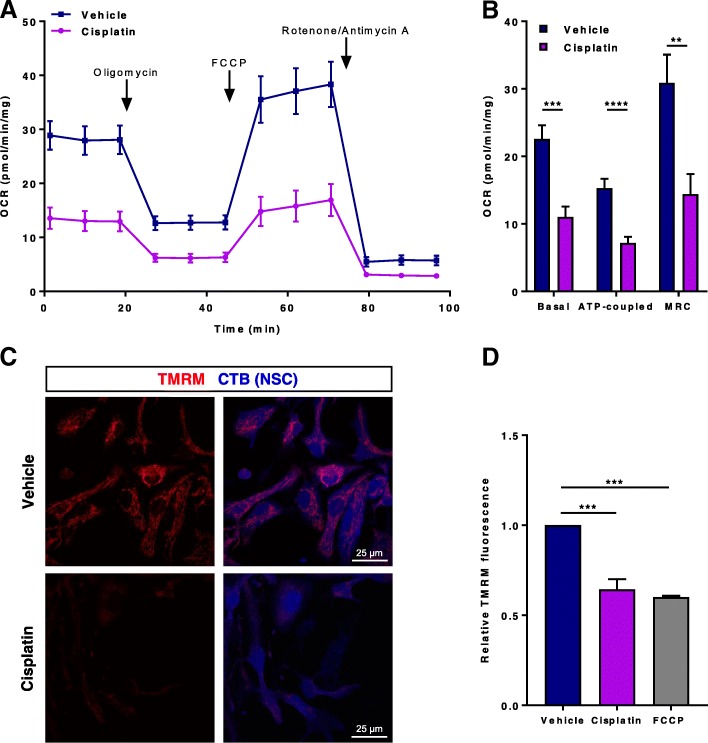
Cisplatin induces NSC mitochondrial dysfunction. Neuronal stem cells (NSCs) were treated with 1 μM cisplatin for 12 h. Oxygen consumption rates (OCR) were analyzed using a Seahorse XFe 24 Analyzer and normalized to protein content (**a**). Mean basal, ATP production-related, and maximum respiratory capacity (MRC) normalized to protein content were calculated (**b**). Results are expressed as means ± SEM of 3 independent experiments. Data were analyzed using Student’s t-test: ** P < 0.01; ***P < 0.001; **** P < 0.0001. NSCs were treated with 1 μM cisplatin for 8 h followed by 17 h in normal medium. Cells were stained with tetramethylrhodamine methyl ester (TMRM) and monitored immediately by live-cell imaging (**c**) or flow cytometry (**d**). NSCs treated with carbonilcyanide p-triflouromethoxyphenylhydrazone (FCCP, 10 μM for 15 min) were used as a positive control. Bar graphs represent mean ± SEM of 3 independent experiments. Data are normalized to mean fluorescence intensity of vehicle-treated cells in each experiment. Data were analyzed using One-way ANOVA followed by Bonferroni’s post-hoc test. *** P ≤ 0.001

To assess whether cisplatin induced mitochondrial damage in NSCs, we measured oxygen consumption rates of NSC using the Seahorse XF24 extracellular flux analyzer (Fig. 2a). NSCs treated with cisplatin showed a marked decrease in basal respiration as well as in oxygen consumption related to ATP production in comparison to control conditions. Furthermore, cisplatin significantly reduced maximal respiration as measured in the presence of FCCP (Fig. 2b).

As a second measure of cisplatin-induced loss of mitochondrial integrity, we assessed mitochondrial membrane potential. NSCs were treated with cisplatin and labeled with the mitochondrial membrane potential-sensitive dye tetramethylrhodamine methyl ester (TMRM). Using live-cell imaging, we observed that cisplatin decreased the TMRM signal, indicating a reduction in mitochondrial membrane potential (Fig. 2c). Quantification of the change in TMRM staining by flow cytometry showed that exposure of NSCs to cisplatin decreased mean TMRM fluorescence intensity by approximately 40%. This decrease in mitochondrial membrane potential was similar to what we observed in response to the mitochondrial uncoupler FCCP, which was used as a positive control (Fig. 2d).

### Mitochondrial transfer from MSC to cisplatin-treated NSC

**Fig. 3:**
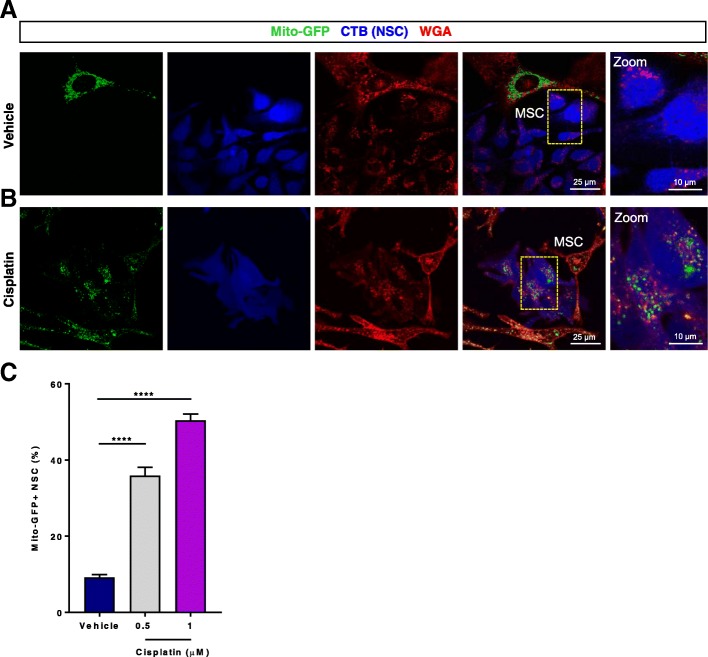
MSCs donate mitochondria to NSCs damaged by cisplatin. Representative confocal images of Neuronal stem cells (NSCs) treated with 1 μM cisplatin or vehicle for 8 h, stained with cell tracker blue (CTB) and subsequently co-cultured for 17 h with mesenchymal stem cells (MSC) transfected with mito-GFP (**a** and **b**) to label the MSC-derived mitochondria. Prior to confocal imaging, co-cultures were stained with wheat germ agglutinin (WGA) AF 594 (**a** and **b**) to reveal cell membranes. GFP-positive mitochondria were detected in NSCs treated with cisplatin (**b**, right panel), indicating mitochondrial transfer. The extent of mitochondrial transfer was quantified by counting the number of NSCs positive for the GFP signal in the co-cultures (**c**). Data are represented as the mean ± SEM of 3 independent experiments and were analyzed using One-way ANOVA followed by Bonferroni’s post hoc-test. **** P < 0.0001

**Fig. 4:**
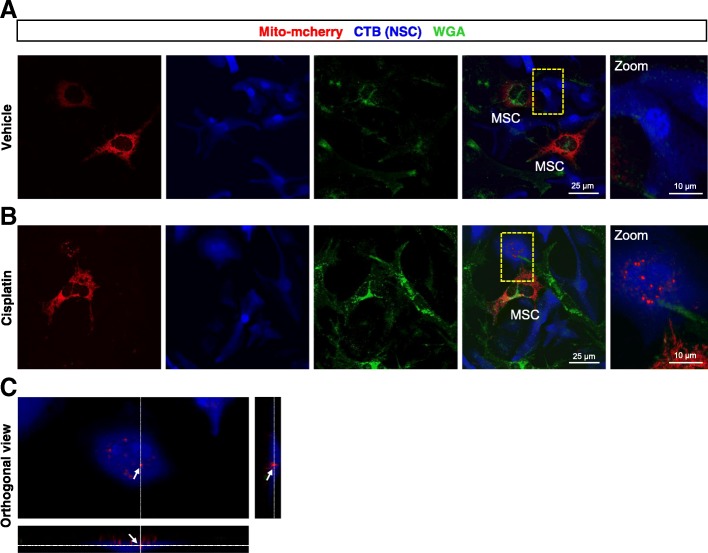
Representative confocal images of Neuronal stem cells (NSCs) and mesenchymal stem cells (MSCs) co-cultures. NSCs were treated with either 1 μM cisplatin or vehicle for 8 h, stained with cell tracker blue (CTB) and co-cultured with MSCs transfected with mito-mcherry plasmid **(a** and **b)** to label the mitochondria. Co-cultures were stained with wheat germ agglutinin (WGA) AF 488 **(a** and **b)** to reveal cell membranes before confocal imaging. mcherry-positive mitochondria were observed in NSCs treated with cisplatin (**b,** right panel**)** showing mitochondrial transfer. **(c)** Orthogonal slice view of NSC containing mcherry-positive mitochondria derived from MSC

Having shown that MSCs can protect NSCs against cisplatin-induced cell death, we hypothesized that the mechanism of the restorative effect of MSCs may involve donation of healthy mitochondria from MSCs to cisplatin-damaged NSCs. To test this possibility, we labeled the mitochondria in the MSCs using mito-GFP (Figs. 3) or mito-mcherry (Fig. 4) plasmids [35]. NSCs were treated with cisplatin or control medium for 8 h, followed by co-culture with MSCs containing the fluorescently tagged mitochondria. Confocal imaging revealed GFP+ or mcherry+ mitochondria in cisplatin-treated NSCs, suggesting mitochondrial transfer from MSCs to NSCs (Figs. 3 and 4). Quantitative assessment of mitochondrial transfer showed that cisplatin dose-dependently increased the percentage of NSC that had received mitochondria from MSCs (Fig. 3c). The orthogonal slice view in Fig. 4c demonstrates that the MSC-derived mitochondria are indeed localized inside the cisplatin-treated NSC.

### MSC normalize mitochondrial membrane potential in cisplatin-treated NSCs

**Fig. 5:**
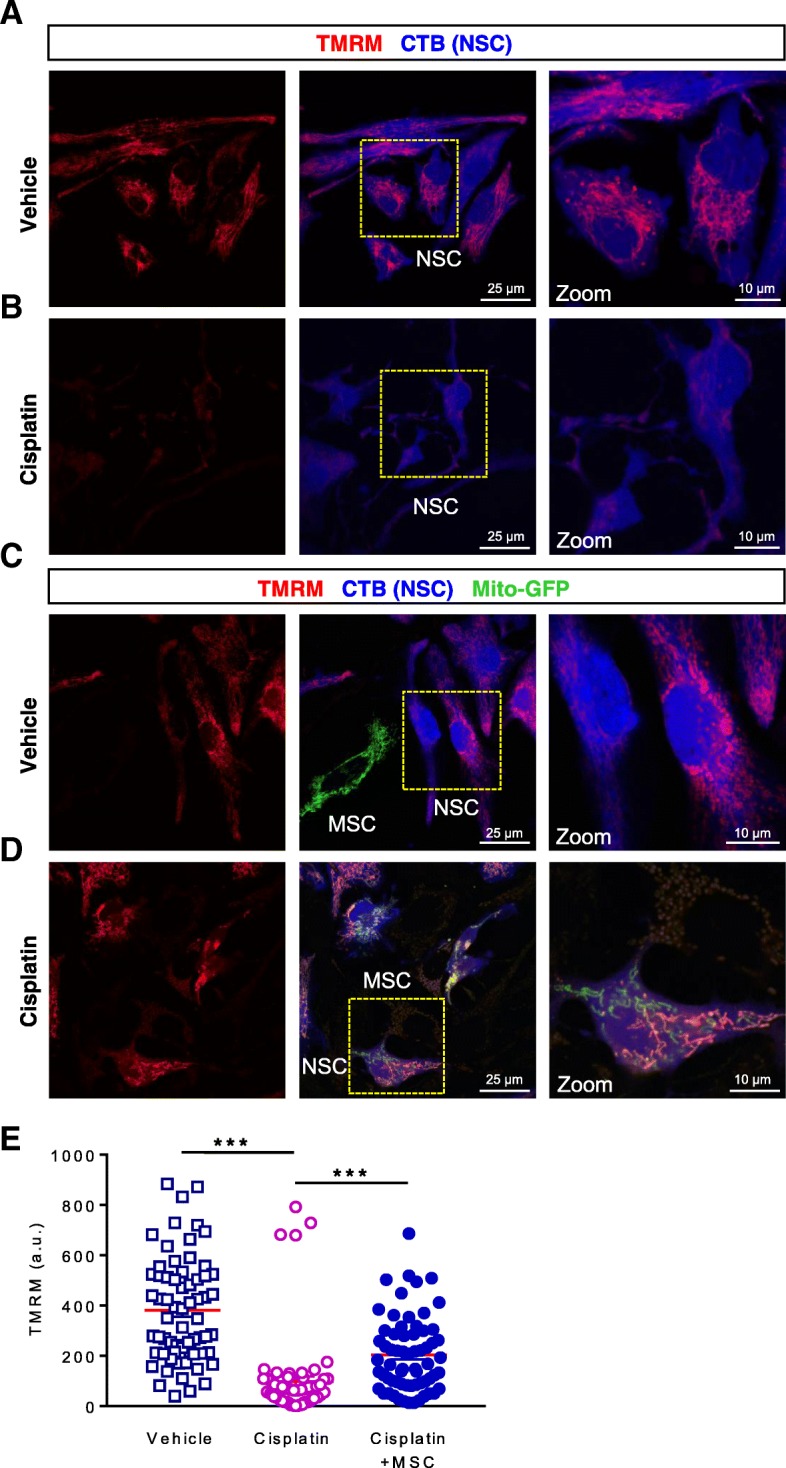
Mitochondrial transfer from MSCs to NSCs damaged by cisplatin restores mitochondrial membrane potential. Neuronal stem cells (NSCs) were treated with vehicle or 1 μM cisplatin for 8 h, stained with cell tracker blue (CTB), and subsequently co-cultured for 17 h with or without mesenchymal stem cells (MSCs) transfected with mito-GFP to label the mitochondria. Co-cultures were stained with tetramethylrhodamine methyl ester (TMRM) and imaged. Confocal images of co-cultures show that NSCs exhibited a bright TMRM signal in control conditions (**a** and **c**) that was markedly reduced after cisplatin treatment (**b**). The mitochondrial membrane potential in cisplatin-treated NSCs was restored after receiving MSC-derived mitochondria labeled in green (**d**). MSCs do not uptake TMRM as well as NSCs (Additional file 1: Figure S1). TMRM fluorescence was quantified in individual NSCs (**e**). *N* = 65 cells were quantified in each group. Data are represented as means ± SEM and were analyzed using One-way ANOVA followed by Dunn’s multiple comparisons test. *** P ≤ 0.001

Cisplatin treatment decreased TMRM staining in NSCs, indicating a reduction in mitochondrial membrane potential (Figs. 2 and 5). We next analyzed whether mitochondrial transfer of MSCs to cisplatin-treated NSCs restored their mitochondrial membrane potential. To that end, we compared TMRM staining in cisplatin-treated NSCs co-cultured with or without mito-GFP-transfected MSCs (Fig. 5). TMRM staining was markedly increased in those NSCs that received mitochondria from the MSCs (as shown by the presence of mito-GFP-labeled mitochondria) in comparison with the NSCs that were negative for mito-GFP (Fig. 5). The latter results indicate that MSC-derived mitochondrial donation restores mitochondrial integrity of NSCs treated with cisplatin.

### Effect of Latrunculin B on transfer of mitochondria and NSC survival

**Fig. 6:**
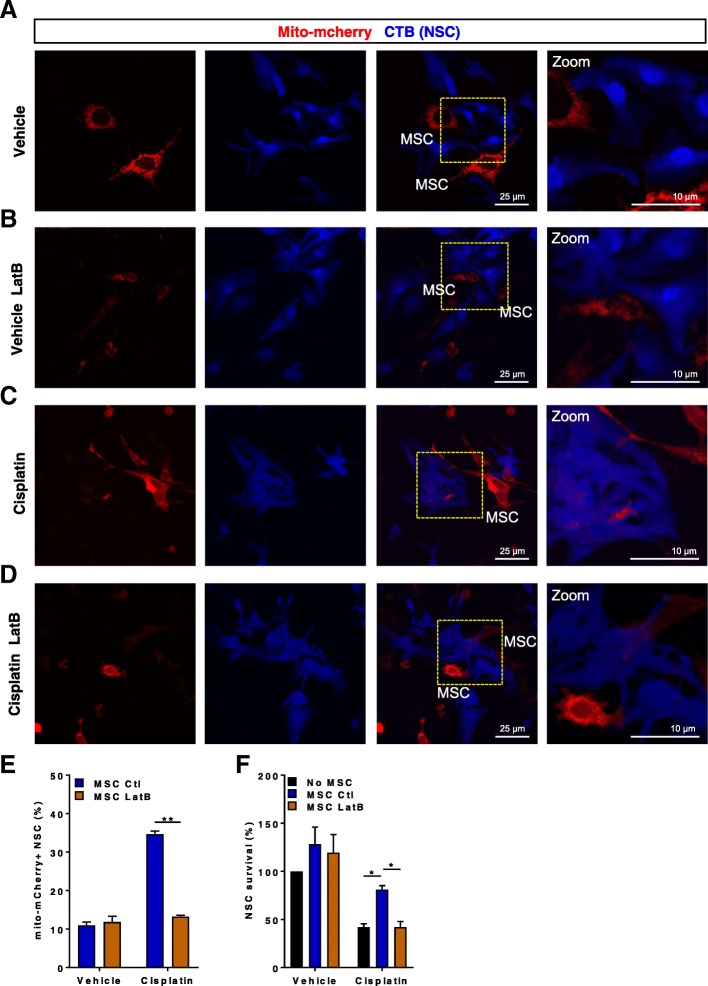
Effect of Latrunculin B on transfer of mitochondria and NSC survival. Representative confocal images of Neuronal stem cells (NSCs) stained with cell tracker blue (CTB) and subsequently co-cultured for 17 h with mesenchymal stem cells (MSC) transfected with mito-mcherry (**a**-**d**) to label the MSC-derived mitochondria. Neuronal stem cells (NSCs) were treated with 1 μM cisplatin or medium for 8 h followed by co-culture with mesenchymal stem cells (MSCs) treated with 2 μm Latrunculin B (MSC LatB, **b** and **d**), control MSC (MSC Ctl, **a** and **c**), or without MSC for 17 h. Mitochondrial transfer (**e**) and NSC survival (**f**) were quantified as in Figs. 1 and 3. Data are represented as means ± SEM of 4 independent experiments and were analyzed using two-way ANOVA followed by Bonferroni’s post-hoc test. ** P < 0.01*;* * *P <* 0.05

Mesenchymal stem cells (MSCs) can transfer mitochondria to other cells via formation of tunneling nanotubes (TNTs) and protrusions [18, 40]. To determine whether mitochondrial donation is crucial for NSC survival, we pre-incubated MSCs with latrunculin B (LatB, an inhibitor of f-actin polymerization) which has been shown to inhibit TNT formation [34].We assessed the effect of LatB on mitochondrial transfer and NSC survival in the co-cultures with MSCs labeled with mito-mcherry. LatB reduced the transfer of mito-mcherry-labeled mitochondria to cisplatin-treated NSCs (Fig. 6d and e). Interestingly, LatB did not affect transfer of mitochondria to untreated NSCs (Figs. 6b and e). Moreover, LatB-treated MSCs were no longer able to promote survival of cisplatin-treated NSCs (Fig. 6f) indicating that mitochondrial transfer by MSC contributes to NSC survival.

### Overexpression of Miro1 in MSC increases mitochondrial transfer and promotes NSC survival

**Fig. 7:**
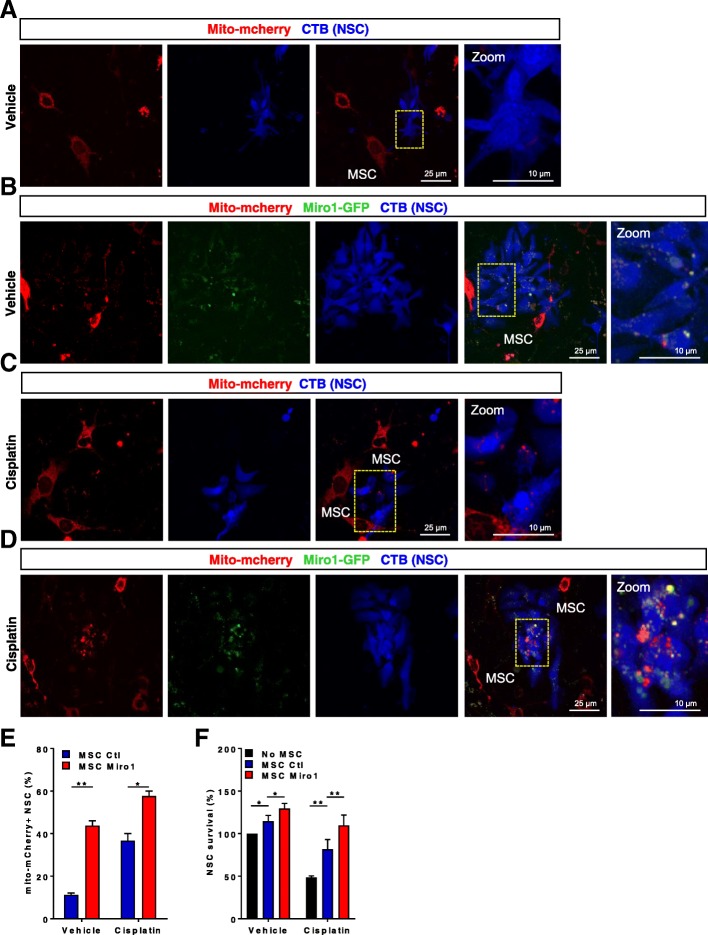
Overexpression of Miro1 in MSCs boosts NSC survival and enhances mitochondrial transfer to injured NSCs. Representative confocal images of Neuronal stem cells (NSCs) stained with cell tracker blue (CTB) and subsequently co-cultured for 17 h with mesenchymal stem cells (MSC) transfected with mito-mcherry (**a**-**d**) and miro1-GFP (**b**, **d**) to label the MSC-derived mitochondria. Neuronal stem cells (NSCs) were treated with 1 μM cisplatin for 8 h and then co-cultured for 17 h with mesenchymal stem cells (MSCs) overexpressing Miro1 GTPase (MSC Miro1), MSCs transfected with empty vector (MSC Ctl), or without MSCs. Mitochondrial transfer (**e**) and survival (**f**) were assessed as in Fig. 6. Data were analyzed by two-way ANOVA followed by Bonferroni’s post-hoc test. ** *P <* 0.01; * *P* < 0.05

Miro-1 is an essential mediator of microtubule-based mitochondrial motility and contributes to mitochondrial transfer between cells [2]. In our search to improve mitochondrial donation by MSCs, we overexpressed Miro1 in MSCs using a mitochondrial Rho GTPase 1 (Miro1)-GFP plasmid. NSCs were treated either with 1 μM cisplatin or vehicle for 8 h, and subsequently co-cultured with or without MSCs overexpressing Miro1 and mito-mcherry or MSCs transfected with control vector and mito-mcherry for 17 h. Overexpression of Miro1 in MSCs increased mitochondrial transfer to NSCs (Figs. 7b, d and e). Moreover, overexpression of Miro1 in MSCs increased their positive effect on survival of cisplatin-treated NSCs (Fig. 7f).

## Discussion

Here we show for the first time that MSCs donate mitochondria to NSCs when damaged by cisplatin in vitro. We also show for the first time that the loss of DCX+ neuronal precursors caused by administration of 2 cycles of cisplatin can be rescued by intranasal administration of MSCs. Transfer of mitochondria from MSCs to NSCs reversed the decrease in mitochondrial membrane potential of the cisplatin-treated NSCs and favored their survival after cisplatin treatment. Blocking MSC-derived mitochondrial transfer by inhibiting actin polymerization eliminated the beneficial effect of MSCs on survival of cisplatin-treated NSCs. Conversely, when mitochondrial transfer was enhanced by overexpression of the Rho-GTPase 1 (Miro1), a mitochondrial motor protein, NSC survival after cisplatin treatment further increased. Collectively, our data support the model that MSCs can transfer mitochondria to damaged NSCs via formation of protrusions and tubular structures to rescue the latter ones after cisplatin treatment.

MSCs have the potential to repair injuries by a variety of mechanisms that range from secretion of paracrine factors and transfer of proteins and RNA to the transfer of organelles such as mitochondria. It is now well recognized that MSCs can transfer mitochondria to various cell types, including epithelial cells, macrophages, cardiomyocytes, neural cells and endothelial cells, in highly toxic or cell-damaging conditions [2, 5, 18, 26, 27, 44, 46]. As mentioned in the introduction, the transfer of mitochondria has also been shown to occur in vivo and may assist in rescuing functional and bioenergetic properties of recipient cells in metabolic need [2, 5, 18, 26, 27, 44, 46].

In our in vitro model of cisplatin-induced neurotoxicity, we observed that cisplatin treatment markedly increased the number of NSCs that received mitochondria from MSCs. This finding implies that cisplatin-treated NSCs express a “danger signal” that prompts MSCs to initiate mitochondrial transfer and therefore promote reparative functions. Several danger signals have been hypothesized to function as inducers of mitochondrial transfer. One possibility is that cells in need release damaged mitochondria and mtDNA that can then be recognized by MSCs via receptors for damage-associated molecular patterns, including toll-like receptors [12]. The actual uptake of damaged mitochondria by MSCs was shown to be crucial for activating MSCs to rescue damaged cardiomyocytes or human umbilical-vein endothelial cells, both in vitro and in vivo [29].

Intercellular communication is crucial for the development and maintenance of tissue growth, differentiation, and regeneration. Cells are capable of establishing direct contact through various types of cell connections, such as formation of cytoplasmic TNTs that enable the transfer of organelles such as mitochondria from one cell to another. Studies have reported that one of the means by which MSCs make contact with injured cells and transfer their mitochondria is via formation of these TNTs [2, 18, 26, 27]. Our finding that LatB reduces mitochondrial transfer indicates that MSCs use TNTs for delivering their mitochondria. The molecular signal inducing the formation of TNTs is still unclear and seems to differ between cell types [40]. Studies conducted in immune cells and HEK293T cells highlight the involvement of the M-Sec pathway, a 73-kDa cytosolic protein also known as tumor necrosis factor α-induced protein 2 or B94, in inducing the membrane protrusion that is one of the first steps in the formation of TNTs [14, 32]. In addition, the tumor suppressor molecule p53 and the Akt/PI3K/mTOR signaling pathway have been shown to play a role in TNT formation in astrocytes [42].

Wang et al. [42] also found that p53 activation is crucial for TNT formation since genetic ablation of p53 prevented formation of TNTs in rat hippocampal co-cultures of astrocytes and neurons. In line with these findings, we have preliminary data indicating that prevention of mitochondrial accumulation of p53 by the mitochondrial protectant pifithrin-μ, decreased the transfer of mitochondria to damaged NSCs in vitro (data not shown). Furthermore, we recently showed that in vivo, cisplatin treatment rapidly induced translocation of p53 to mitochondria in the brain. Inhibition of p53 translocation to mitochondria prevented cisplatin-induced mitochondrial dysfunction of neuronal synaptosomes [7]. Therefore, we propose that translocation of p53 to the mitochondria of the damaged acceptor cell is part of the complex that confers a danger signal to the donor cell and prompt MSCs to transfer healthy mitochondria to NSCs.

Mitochondrial transfer between donor and acceptor cells requires mitochondrial movement along the actin cytoskeleton of the cells. This movement of mitochondria is regulated by the mitochondrial membrane Rho-GTPase 1 (Miro1), which binds the cytoplasmic adaptor protein milton and kinesin heavy chain through its cytoplasmic domains, thereby connecting mitochondria to the actin cytoskeleton. We show that Miro1 overexpression in MSCs increased the transfer of mitochondria and enhanced the rescue potential of MSCs when co-cultured with cisplatin-damaged NSCs. Ahmad et al. [2] demonstrated in an in vitro model using lung epithelial cells that genetic manipulation of MSCs to overexpress Miro1 resulted in higher efficiency of transfer from MSCs to epithelial cells. Interestingly, intravenous administration of MSCs overexpressing Miro1 improved the efficacy of MSCs to reduce rotenone-induced lung injury [2]. The mechanism of action of Miro1 is complex but Schuler et al. recently showed using mouse embryonic fibroblasts that increasing Miro1 in MSCs may lead to better positioning of mitochondria at the leading edge of the cytoplasm instead of in the perinuclear area, allowing higher cytoplasmic energy redistribution and thereby favoring protrusion formation and MSC migration [36].

We do not yet know how mitochondria of donor origin rescue mitochondrial health and promote survival of the damaged acceptor NSCs. One possibility is that the donor mitochondria fuse with mitochondria in the acceptor cell, thereby restoring bioenergetic efficiency. Another potential mechanism is that the acceptor cell discards the damaged mitochondria and instead hijacks the donated mitochondria for energy production. Future research is needed to investigate how the MSC-derived mitochondria communicate with the acceptor cell cellular machinery to foster cellular health.

MSCs are becoming excellent candidates for regenerative strategies to restore brain damage after traumatic brain injury, stroke, hypoxia–ischemia, and neurodegenerative disorders like Alzheimer disease [9–11, 24, 25, 45]. We recently observed that nasal administration of MSCs can restore the cognitive deficits that arise after cisplatin treatment [6, 16]. In relation to this finding, it is of interest that we observed that cisplatin-induced loss of DCX+ neuroblasts was reversed by intranasal administration of MSCs as well. We propose that MSC-mediated transfer of mitochondria to NSCs reverses the neurotoxic effects of cisplatin, thereby facilitating cognitive processes.

The improvement in mitochondrial integrity and survival of NSCs in the presence of MSCs is likely not limited to the transfer of mitochondria alone. MSCs are known to favorably influence the growth factor milieu, which could also aid in rescuing cisplatin-damaged NSCs [38]. However, when we blocked the transfer of mitochondria to NSCs using LatB, which depolymerizes F-actin and thereby inhibits TNT formation, we no longer observed the positive effect of MSCs on the NSC survival in vitro. These findings indicate that mitochondrial transfer plays a major role in the rescue of NSCs damaged by cisplatin.

In conclusion, we propose that the transfer of healthy mitochondria is an important mechanism underlying the regenerative effects of MSCs in the brain.

## Additional file

Additional file 1: **Figure S1.** Representative images of MSC harboring GFP labeled mitochondria and TMRM staining. MSCs were transfected with mito-GFP, stained with TMRM and imaged. MSCs do not exhibit a strong TMRM signal as compared to NSCs. (PDF 106 kb)

## Abbreviations

ATP: Adenosine triphosphate
CTB: Cell Tracker Blue fluorescent probe
DCX: Doublecortin
DG: Dentate Gyrus
FCCP: Carbonilcyanide p-triflouromethoxyphenylhydrazone
GFAP: Glial fibrillary acidic protein
GFP: Green fluorescent protein
LatB: Latrunculin B
Miro1: Mitochondrial Rho GTPase 1
MSC: Mesenchymal stem cell
NSC: Neuronal stem cell
SVZ: Subventricular Zone
TMRM: Tetramethylrhodamine methyl ester
TNT: Tunneling nanotube
WGA: Wheat germ agglutinin
