# Supplementary figures and images for: Mitochondrial transfer from mesenchymal stem cells to neural stem cells protects against the neurotoxic effects of cisplatin
Source: Acta Neuropathol Commun. 2018 Dec 12;6:139. doi: 10.1186/s40478-018-0644-8 (PMC6292021; doi:10.1186/s40478-018-0644-8)

Figure S1

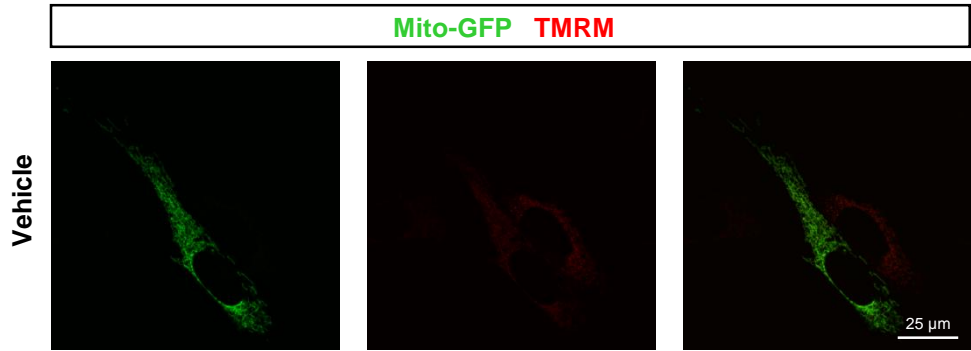

Supplement: Supplementary file 1 — Figure S1. Representative images of MSC harboring GFP labeled mitochondria and TMRM staining. MSCs were transfected with mito-GFP, stained with TMRM and imaged. MSCs do not exhibit a strong TMRM signal as compared to NSCs. (PDF 106 kb) [file 40478_2018_644_MOESM1_ESM.pdf]
